# Supplementary figures and images for: HOXA10-TWIST2 antagonism drives partial epithelial-to-mesenchymal transition for embryo implantation
Source: Cell Death Discov. 2025 Nov 10;11:516. doi: 10.1038/s41420-025-02799-w (PMC12603138; doi:10.1038/s41420-025-02799-w)

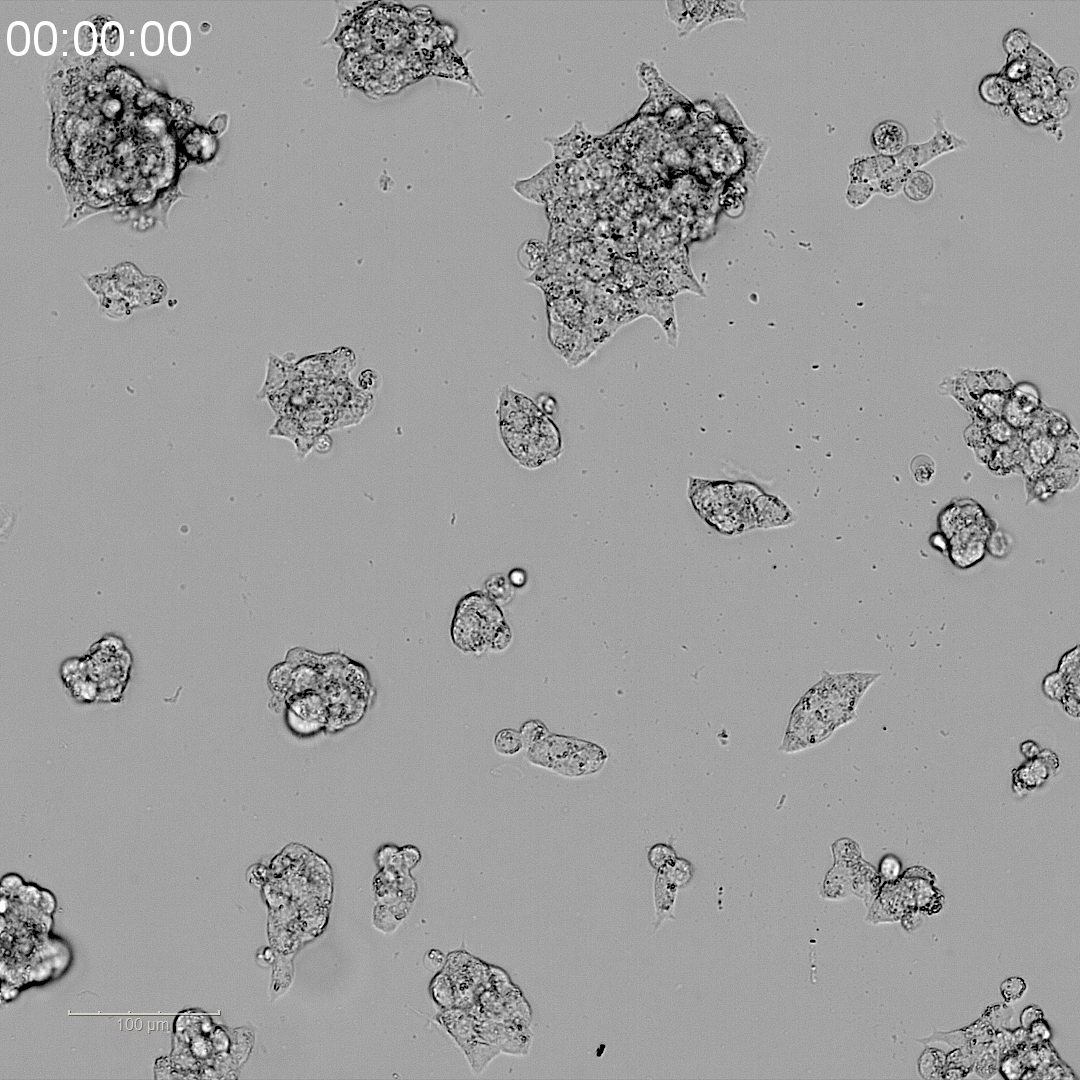

Supplement: Supplementary file 1 — Supplementary Movie 1 [file 41420_2025_2799_MOESM1_ESM.gif]

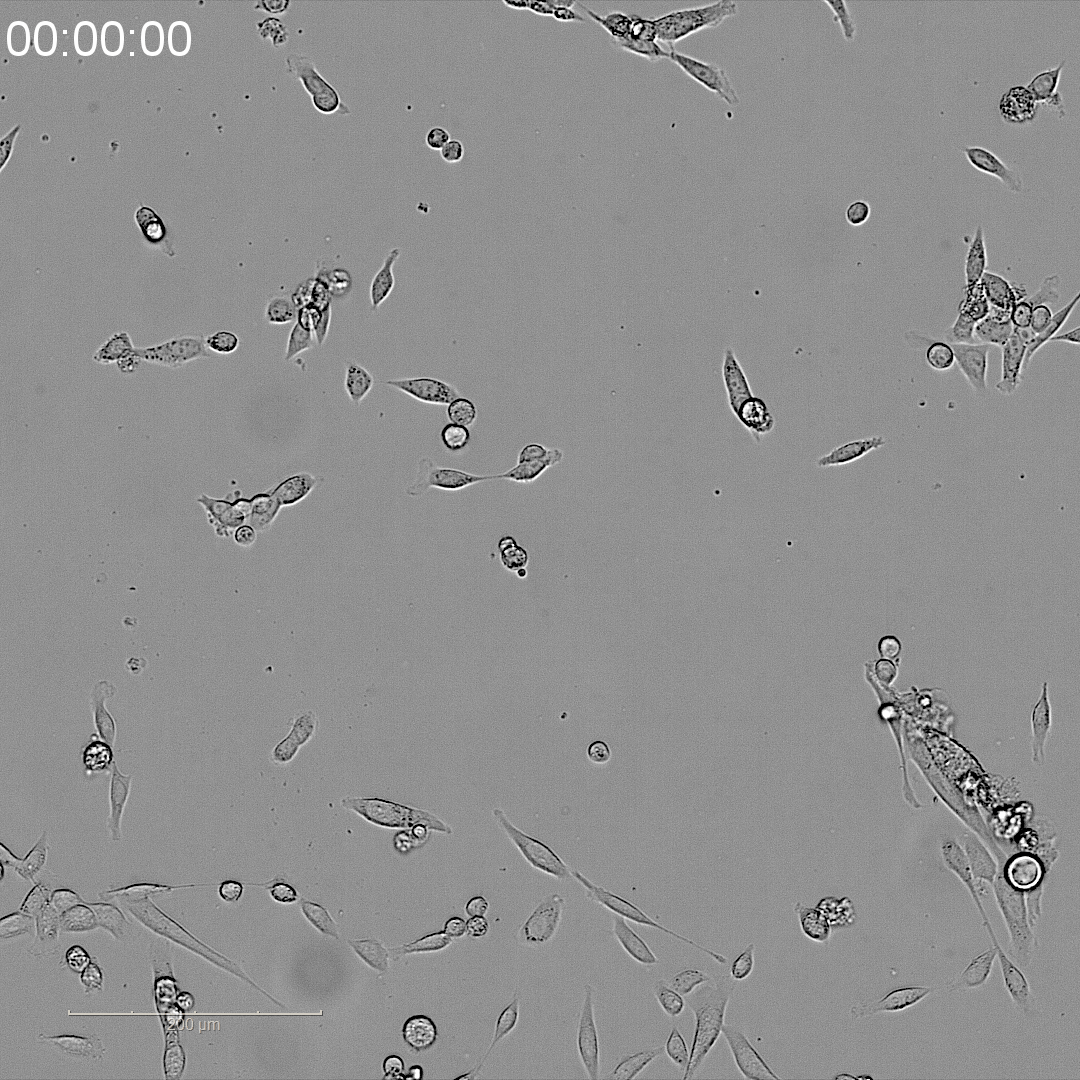

Supplement: Supplementary file 2 — Supplementary Movie 2 [file 41420_2025_2799_MOESM2_ESM.gif]

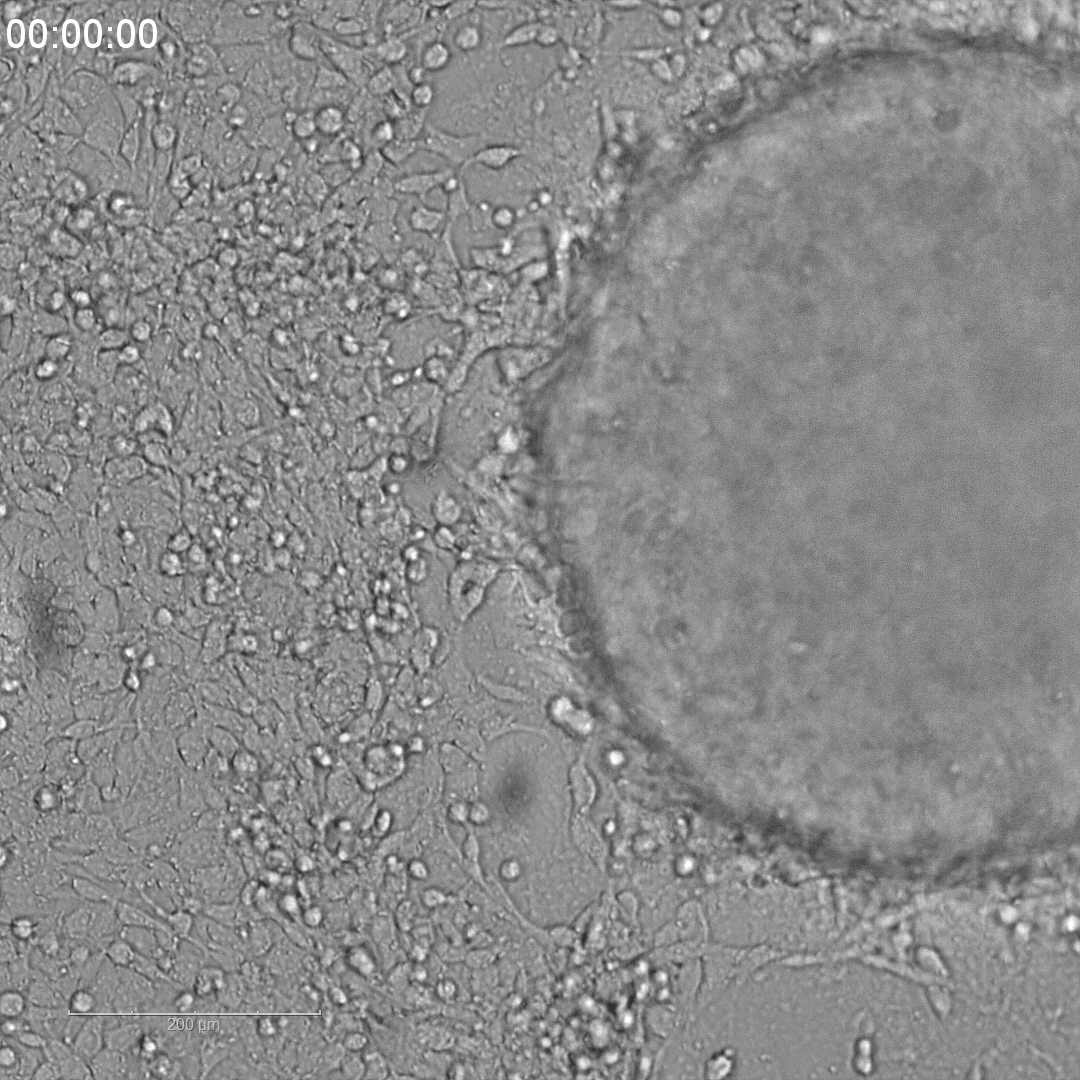

Supplement: Supplementary file 3 — Supplementary Movie 3 [file 41420_2025_2799_MOESM3_ESM.gif]
