# Supplementary material for: HOXA10-TWIST2 antagonism drives partial epithelial-to-mesenchymal transition for embryo implantation
Source: Cell Death Discov. 2025 Nov 10;11:516. doi: 10.1038/s41420-025-02799-w (PMC12603138; doi:10.1038/s41420-025-02799-w)
Supplement: Supplementary file 4 — Supplementary Materials and Methods [file 41420_2025_2799_MOESM4_ESM.docx]

**Supplementary Materials and Methods**

**1. Immunohistochemistry**

Immunohistochemistry was performed as described^28^; Briefly, paraffin embedded sections were deparaffinized in xylene and rehydrated in grades of alcohol. Antigen was unmasked using Tris EDTA buffer (10 mM, pH 9) at 90 °C. The sections were blocked using 1% donkey serum (Jackson Immunology) for 1h. Further sections were probed with a primary antibody (HOXA10) for overnight. Next day sections were washed and incubated with a biotinylated secondary antibody followed by streptavidin-HRP (ABC kit Santa Cruz Biotechnology). 3, 3′-diaminobenzidine (DAB) (Sigma -Aldrich) was used as a chromogen and hydrogen peroxidase as a substrate for the detection. Sections were counterstained with haematoxylin and mounted in DPX. Slides were viewed under a bright field microscope (Olympus) and representative areas were photographed. "Fiji" version of ImageJ software was used to quantification of HOXA10 expression

.

**2. RT-PCR**

Total RNA from cells were extracted using Trizol reagent (Invitrogen) as described previously^28^; and reverse-transcribed to cDNA (cDNA reverse transcriptase kit, Applied Biosystems). Real time PCR was performed using the CFX-96 thermal cycler (Bio-Rad) using SYBR green chemistry (Bio-Rad). The annealing temperature was optimized for each gene. Gene expression was normalized to the levels of 18S, and fold change was calculated.

| Gene Name | Primer sequence  5’-3’ |
| --- | --- |
| *HOXA10* | 5'-GCCCCTTCCGAGAGCAGAAAA-3'  5'-AGGTGGAGCCTGCGGCTAATCTCTA-3' |
| *CDH1* (E-Cadherin) | 5'-GAACAGCACGTACACAGCCCT-3'  5'-GCAGAAGTGTCCCTGTTCCAG-3' |
| *CDH2* (N-Cadherin) | 5'-GACGGTTCGCCATCCAGAC-3'  5'-TCGATTGGTTTGACCACGG-3' |
