# Supplementary material for: HOXA10-TWIST2 antagonism drives partial epithelial-to-mesenchymal transition for embryo implantation
Source: Cell Death Discov. 2025 Nov 10;11:516. doi: 10.1038/s41420-025-02799-w (PMC12603138; doi:10.1038/s41420-025-02799-w)
Supplement: Supplementary file 5 — Supplementary Movie Legends [file 41420_2025_2799_MOESM5_ESM.docx]

**Supplementary Movie legends:**

**Movie 1:**  Representative phase contrast timelapse of control endometrial epithelial cells (RL95). Cells were imaged every 30 min for 24h using the Operetta CLS High-Content Analysis system equipped with 37 °C, 5% CO2 environmentally controlled chamber (Revvity). Total elapsed time from first image is indicated at top left

**Movie 2:**  Representative phase contrast timelapse of *HOXA10*KD endometrial epithelial cells (RL95). Cells were imaged every 30 min for 24h using the Operetta CLS High-Content Analysis system equipped with 37 °C, 5% CO2 environmentally controlled chamber (Revvity). Total elapsed time from first image is indicated at top left.

**Movie 3:**  Representative phase contrast timelapse of *HOXA10*KD cells co-incubated with trophoblast spheroid (HRT8/SV-neo). Cells were imaged every 30 min for 12h using the Operetta CLS High-Content Analysis system equipped with 37 °C, 5% CO2 environmentally controlled chamber (Revvity). Total elapsed time from first image is indicated at top left.
