# Supplementary material for: HOXA10-TWIST2 antagonism drives partial epithelial-to-mesenchymal transition for embryo implantation
Source: Cell Death Discov. 2025 Nov 10;11:516. doi: 10.1038/s41420-025-02799-w (PMC12603138; doi:10.1038/s41420-025-02799-w)
Supplement: Supplementary file 6 — SupplementaryTables [file 41420_2025_2799_MOESM6_ESM.docx]

|  | Role In Embryo Implantation |
| --- | --- |
| Estrogen receptor | Estrogen is a critical determinant that specifies the duration of the window of uterine receptivity for implantation ^83,84^. |
| KLF5 | Klf5 is critical to making the uterine luminal epithelium conducive to blastocyst implantation and growth. Epithelium is retained in Klf5 deleted females past the WOI and Decidualization in impaired^85^. |
| FOXA2 | FOXA2 is a critical regulator of uterine gland function, embryo implantation, and pregnancy establishment. Blastocyst attachment in adult FOXA2-deficient uteri is impaired due to stromal cell decidualization impairment. Hoxa10 expression was increased in the implantation sites of FOXA2 deleted mice^86^. |
| KLF4 | KLF4 promotes decidualization of human endometrial stromal cells by regulating their autophagy level. In adenomyotic human endometrial stromal cells decidualization id impaired, overexpression of KLF4 significantly reversed the decreased secretion of decidualization PRL^87^. |
| KLF9 | BTEB1, the transcription factor of KLF9, by regulating Stromal progesterone receptor expression and transactivation, participates in the paracrine control of luminal epithelium proliferation by PGR and thus is important for establishment of a receptive uterus critical for successful implantation^88^. |
|  | HOXA10-binding sites in the KLF9 promoter directly regulate KLF9 expression. Together they likely regulate progesterone action in endometrial epithelial cells^89^. |
| FOXO1 | Uterine ablation of Foxo1 using the progesterone receptor Cre (PgrCre) mouse model resulted in infertility due to altered epithelial cell polarity and apoptosis, preventing the embryo from penetrating the luminal epithelium. Foxo1 knocked Down mice indicated that endometrial FOXO1 was required for the temporal permeability of the LE^90^. |
| SMADs | Uterine epithelial BMP/SMAD1/5 signalling is essential during early pregnancy and SMAD1/5 epithelial-specific deletion has detrimental effects on stromal cell decidualization and pregnancy development. In Smad1/5 knocked down mice implantation failed with decrease in COX2 expression and FOXO cytoplasmic miss localization^91^. |
|  | Uteri from Smad1, smad5, smad4 and Amhr2 conditionally Knocked down females exhibit multiple defects in stroma, epithelium, and smooth muscle layers and fail to assemble a closed uterine lumen upon embryo implantation, with defective uterine decidualization that led to pregnancy loss at early to mid-gestation^92^. |
|  | Inhibition of SMAD2/3 signalling disrupts organoid morphology, increases the glandular and secretory cell markers, FOXA2 and MUC1, and alters the genome-wide distribution of SMAD4. TGFβ family signalling via SMAD2/3 controls signalling networks which are integral for endometrial cell regeneration and differentiation^93^. |
|  | TGFβ family signalling via SMAD2/3 controls signaling networks which are integral for endometrial cell regeneration and differentiation. Mechanistic studies in endometrial organoids show that inhibition of SMAD2/3 signalling disrupts organoid morphology, increases the glandular and secretory cell markers, FOXA2 and MUC1, and alters the genome-wide distribution of SMAD4^94^. |
|  | Inhibiting SMAD3 on day 3 of pregnancy in mice showed reduction in IGFBP-1 and decreased number of implanted embryos^95^. |
| STAT3 | STAT3 activation in luminal epithelium, regulates epithelial cell-cell junctions, polarity, and function during the receptive phase and also promotes stromal proliferation at the time of decidualization via paracrine growth regulatory signals originating in the epithelium^96^. |

**Supplementary. Table 1. List of motifs co-enriched in the HOXA10 cistrome and have known roles in implantation.**

| Pathway | Role in embryo implantation  **Supplementary. Table 2. List of Signaling pathways and its role in embryo implantation** |
| --- | --- |
| Hippo signaling | The study demonstrates the expressions of pYAP, YAP, TEAD1, and CTGF which are members of the Hippo signaling pathway, in the uterus of mice during the peri-implantation phase. The Hippo signaling pathway shows dynamic changes during the peri-implantation period and is involved in both implantation and decidualization^97,98^. |
| AMPK | AMPK activity is a key mediator of various steroid hormone-dependent processes during pregnancy establishment, including decidualization, uterine receptivity, and epithelial cell proliferation^99^. |
| TGFBeta | Active TGF-B1, available from its latent complex, triggers SMAD3 which is crucial for embryo implantation. Loss of active TGF-B1 affects the development of blastocyst and endometrium receptivity that leads to the reduction in fetus number^100^. |
|  | Expression levels of some immunomodulatory cytokines in endometrium are significantly increased even before the embryo invades the endometrium. The endometrial expression of TGFβ2, TGFβ2 receptor, PP14 and IL-6 were significantly up-regulated (p < 0.05) in pregnant animals as compared to non-pregnant animals, whereas the expression of LIF and its receptor remained unaltered in pregnant animals^101^. |
| VEGF | VEGF plays a crucial role in mediating the increase in estrogen-induced uterine vascular permeability, and it is essential for implantation^102^. |

**Supplementary. Table 3. List of antibodies and their optimized dilutions used in this study**

| Primary Antibody | Company |  | Catalogue | Dilution |
| --- | --- | --- | --- | --- |
| HOXA10 | GenScript,Piscataway, NJ, USA |  | customized | 1:50 |
| HOXA10 | Biomatik,Ontario, Canada |  | CAE03449 | 1:200 |
| E-Cad | Abcam, Cambridge, UK |  | ab15148 | 1:50 |
| N-Cad | Abcam |  | ab18203 | 1:100 |
| KRT8 | Abcam |  | ab154301 | 1:250 |
| TWIST2 | NovusBiologicals, Centennial, CO, USA |  | NBP2-56209 | 1:100 |
| F-actin | Biotium, Fremont, CA, USA |  | Phalloidin, CF®568 | 1:50 |
| Goat anti-rabbit IgG Alexa 568 | Invitrogen, Thermo Fisher Scientific, Waltham, MA, USA |  | A11011 | 1:1000 |

**Supplementary. Table 4. TWIST2 esiRNA and Control esiRNA details**


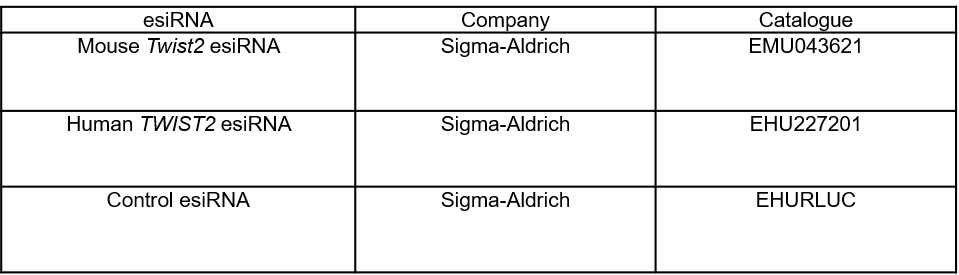
**Supplementary Table References:**

83. Ma W ge, Song H, Das SK, Paria BC, Dey SK. Estrogen is a critical determinant that specifies the duration of the window of uterine receptivity for implantation. *Proc Natl Acad Sci U S A* 2003; **100**: 2963.

84. Okur HSK, Das A, Taylor RN, Bagchi IC, Bagchi MK. Roles of estrogen receptor-α and the coactivator MED1 during human endometrial decidualization. Mol Endocrinol 2016; 30: 302–313.

85. Sun X, Zhang L, Xie H, Wan H, Magella B, Whitsett JA *et al.* Kruppel-like factor 5 (KLF5) is critical for conferring uterine receptivity to implantation. *Proc Natl Acad Sci U S A* 2012; **109**: 1145–1150.

86. Kelleher AM, Peng W, Pru JK, Pru CA, Demayo FJ, Spencer TE. Forkhead box a2 (FOXA2) is essential for uterine function and fertility. *Proc Natl Acad Sci U S A* 2017; **114**: E1018–E1026.

87. Mei J, Sheng X, Yan Y, Cai X, Zhang C, Tian J *et al.* Decreased Krüppel-like factor 4 in adenomyosis impairs decidualization by repressing autophagy in human endometrial stromal cells. *BMC Mol cell Biol* 2022; **23**. doi:10.1186/S12860-022-00425-6.

88. Velarde MC, Van Geng, Eason RR, Simmen FA, Simmen RCM. Null Mutation of Krüppel-Like Factor9/Basic Transcription Element Binding Protein-1 Alters Peri-Implantation Uterine Development in Mice. *Biol Reprod* 2005; **73**: 472–481.

89. Du H, Sarno J, Taylor HS. HOXA10 Inhibits Kruppel-Like Factor 9 Expression in the Human Endometrial Epithelium. *Biol Reprod* 2010; **83**: 205–211.

90. Vasquez YM, Wang X, Wetendorf M, Franco HL, Mo Q, Wang T *et al.* FOXO1 regulates uterine epithelial integrity and progesterone receptor expression critical for embryo implantation. *PLoS Genet* 2018; **14**: e1007787.

91. Tang S, Cope DI, Vasquez YM, Monsivais D. BMP/SMAD1/5 Signaling in the Endometrial Epithelium Is Essential for Receptivity and Early Pregnancy. *Endocrinology* 2022; **163**: 1–15.

92. Rodriguez A, Tripurani SK, Burton JC, Clementi C, Larina I, Pangas SA. SMAD Signaling Is Required for Structural Integrity of the Female Reproductive Tract and Uterine Function During Early Pregnancy in Mice. *Biol Reprod* 2016; **95**:44

93. Kriseman ML, Tang S, Liao Z, Jiang P, Parks SE, Cope DI *et al.* SMAD2/3 signaling in the uterine epithelium controls endometrial cell homeostasis and regeneration. *Commun Biol* 2023; **6**: 261.

94. Zhao KQ, Lin HY, Zhu C, Yang X, Wang H. Maternal Smad3 deficiency compromises decidualization in mice. *J Cell Biochem* 2012; **113**: 3266–3275.

95. Li J, Dong X yuan, Yang P wen, Yang S lin, Hu D, Zhang H wang *et al.* Activation of Uterine Smad3 Pathway Is Crucial for Embryo Implantation. *Curr Med Sci* 2019; **39**: 997–1002.

96. Pawar S, Starosvetsky E, Orvis GD, Behringer RR, Bagchi IC, Bagchi MK. STAT3 regulates uterine epithelial remodeling and epithelial-stromal crosstalk during implantation. *Mol Endocrinol* 2013; **27**: 1996–2012.

97. Golal E, Balci CN, Ustunel I, Acar N. The investigation of hippo signaling pathway in mouse uterus during peri-implantation period. *Arch Gynecol Obstet* 2023; **307**: 1795–1809.

98. Moldovan GE, Massri N, Vegter EL, Pauneto-Delgado IN, Burns GW, Joshi N et al. YAP1 and WWTR1 are required for murine pregnancy initiation. Reproduction 2025; 169: e240355.

99. Griffiths IV RM, Pru CA, Behura SK, Cronrath AR, McCallum ML, Kelp NC *et al.* AMPK is required for uterine receptivity and normal responses to steroid hormones. *Reproduction* 2020; **159**: 707.

100. Maurya VK, Jha RK, Kumar V, JoshiAnubha, Chadchan S, Mohan JJ *et al.* Transforming growth factor-beta 1 (TGF-B1) liberation from its latent complex during embryo implantation and its regulation by estradiol in mouse. *Biol Reprod* 2013; **89**: 84.

101. Rosario GX, Sachdeva G, Manjramkar DD, Modi DN, Meherji PK, Puri CP. Endometrial expression of immunomodulatory cytokines and their regulators during early pregnancy in bonnet monkeys (Macaca radiata). *Hum Reprod* 2005; **20**: 3039–3046.

102. Rockwell LC, Pillai S, Olson CE, Koos RD. Inhibition of Vascular Endothelial Growth Factor/Vascular Permeability Factor Action Blocks Estrogen-Induced Uterine Edema and Implantation in Rodents. *Biol Reprod* 2002; **67**: 1804–1810.
