# Supplementary material for: HOXA10-TWIST2 antagonism drives partial epithelial-to-mesenchymal transition for embryo implantation
Source: Cell Death Discov. 2025 Nov 10;11:516. doi: 10.1038/s41420-025-02799-w (PMC12603138; doi:10.1038/s41420-025-02799-w)
Supplement: Supplementary file 7 — Supplementary Figure 1-9 [file 41420_2025_2799_MOESM7_ESM.pdf]

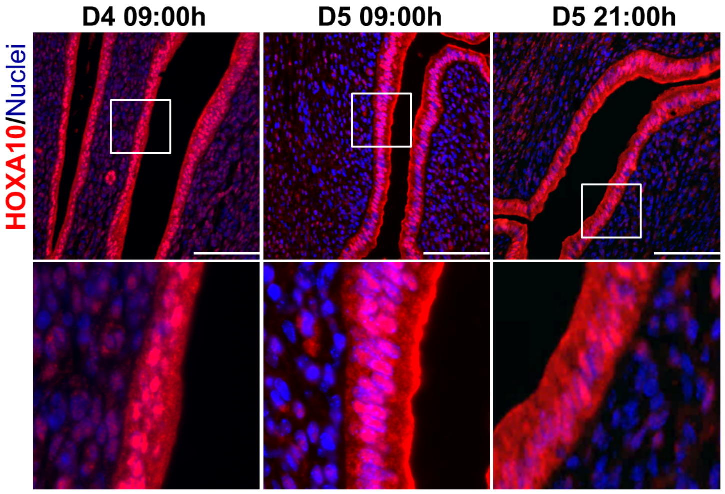

Supplementary Fig.1: Immuno-localization of HOXA10 at the inter-implantation site of mouse endometrium

Selected area is boxed and zoomed in below image. Scale bar = 100μm, n=3

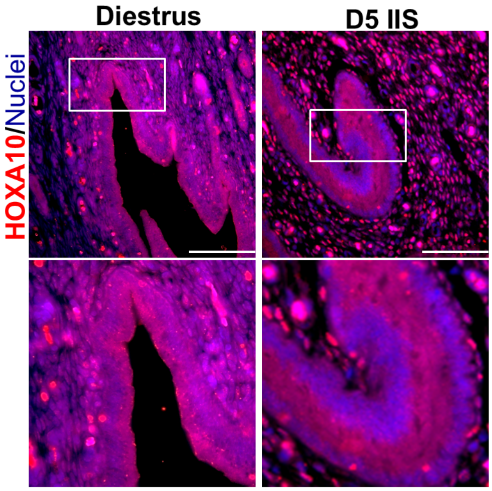

Supplementary Fig.2: Immuno-localization of HOXA10 in Diestrus stage and inter-implantation site (IIS) of Day 5 (D5) hamster endometrium

Selected area is boxed and zoomed in below image. Scale bar = 100µm, n=3

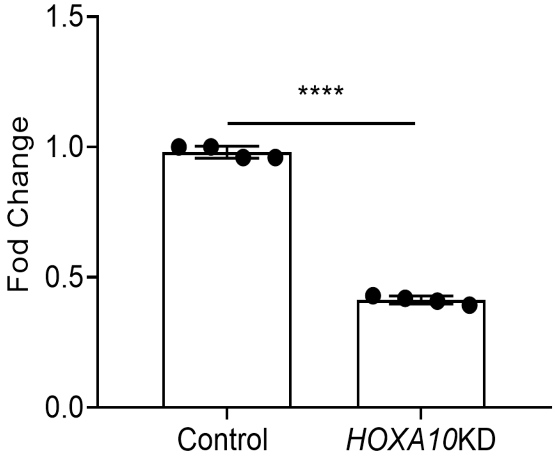

Supplementary Fig.3. Validation of *HOXA10* knockdown in endometrial epithelial cells (RL95-2)
mRNA levels of *HOXA10* (normalize to 18s) in RL95-2 cells stably expressing scrambled shRNA (Control) and *HOXA10* shRNA (*HOXA10KD)*. Y-axis is fold change where values obtained from control cells was taken as 1. Data is the mean ± SD for the Four independent replicates. **** indicates significant difference as compared to control ($p < 0.0001$)

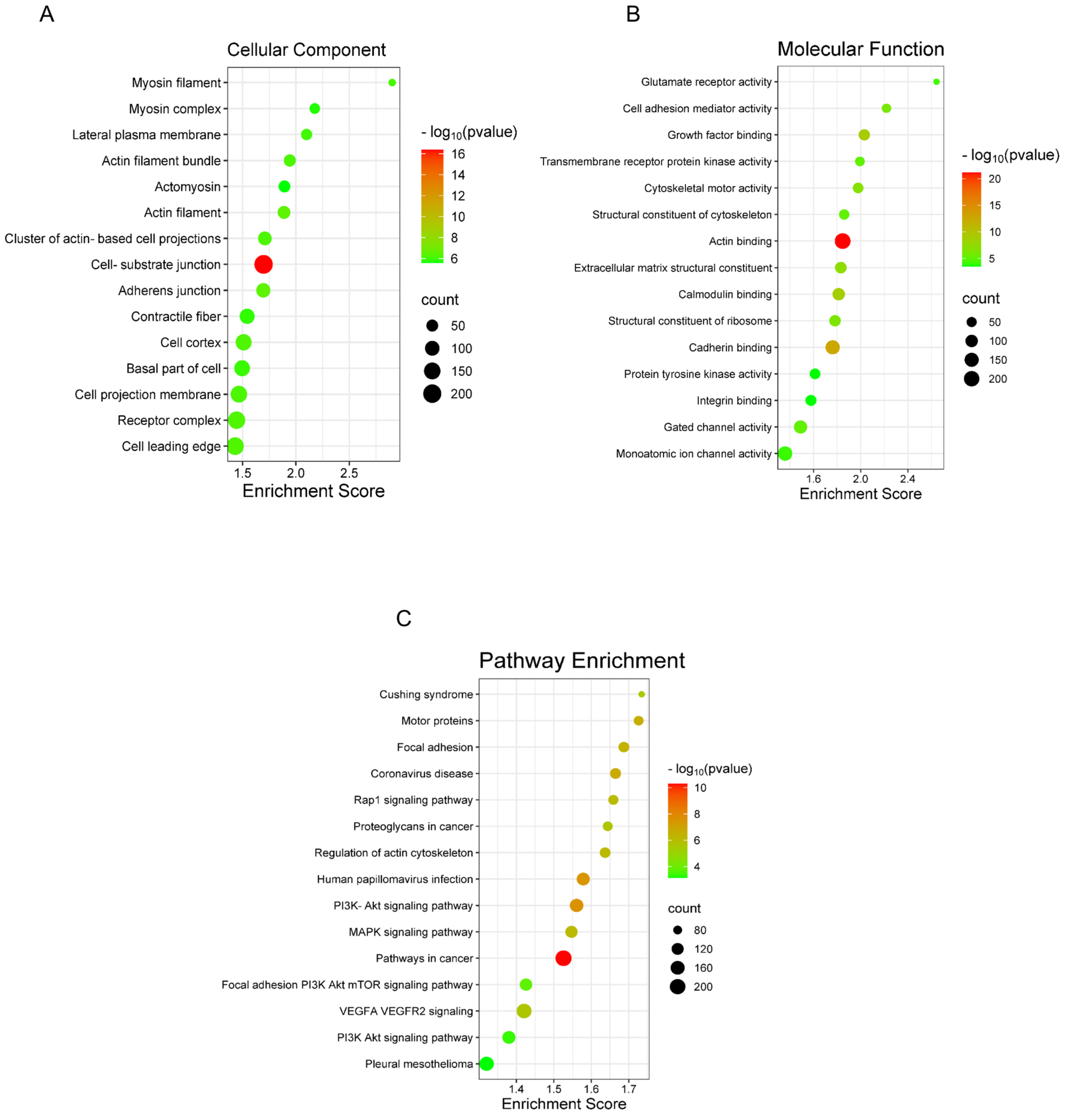

Supplementary Fig.4: Gene Ontology and Pathway Enrichment Analysis of the DEGs in the *HOXA10* knockdown in human endometrial epithelial cells (RL95-2) This figure depicts the results of Gene Ontology (GO) enrichment analysis for **(A)** cellular component; **(B)** molecular functions, **(C)** pathways. Enrichment scores (x-axis) indicate the statistical significance of the enrichment for each term or pathway. Bubble size represents the number of genes associated with each term, and color indicates the -log10(p-value).

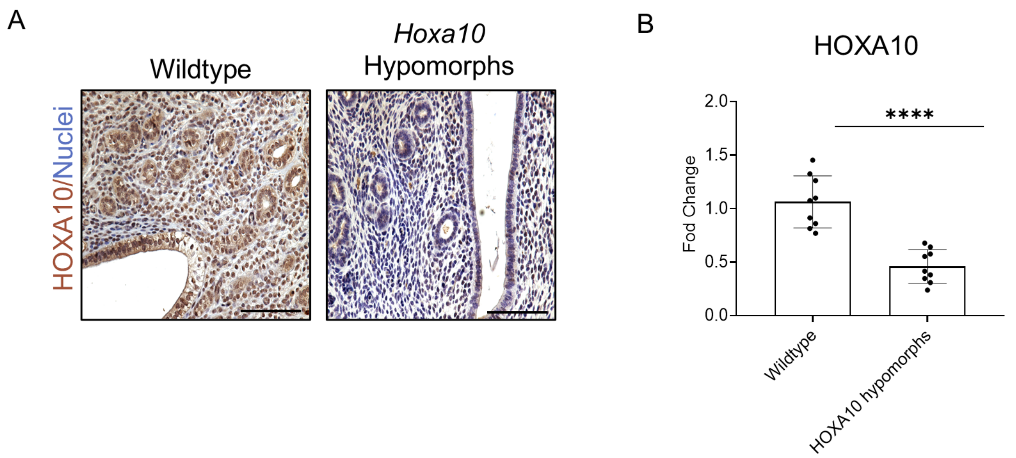

Supplementary Fig.5: Expression of HOXA10 in the uteri of Mice transgenic for shRNA against *Hoxa10* (*Hoxa10* hypomorphs). (A) Immunohistochemistry of HOXA10 in wildtype and *Hoxa10* hypomorphs in diestrus stage mouse endometrium. **(B)** Graph represent intensity of HOXA10 immunostaining. Values on Y-axis are fold change where the mean value of controls was taken as 1. Scale bar = 100μm. ****in Graph represent statistically significant (p<0.0001) with mean ±SD values (n=3) are shown.

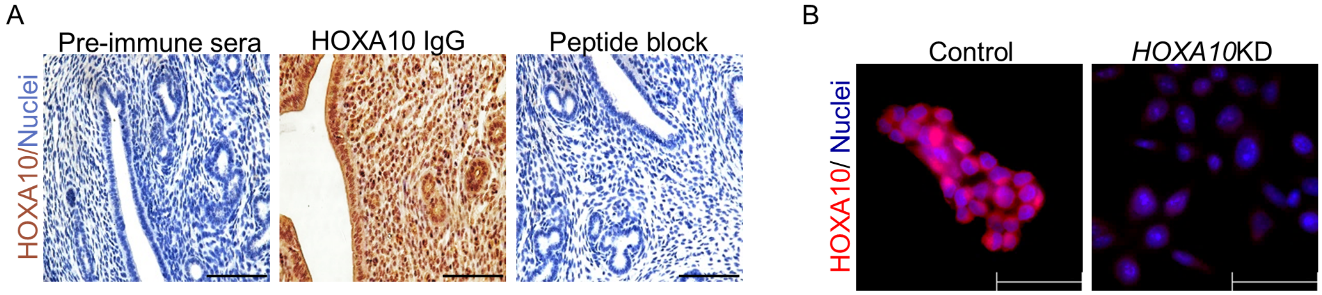

C CUT&RUN of HOXA10 in RL95-2 cells

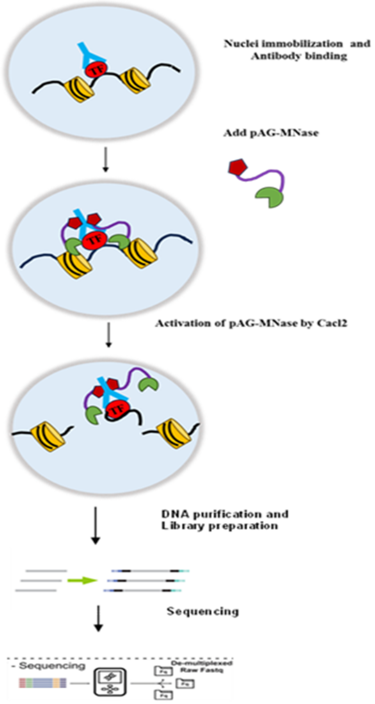

HOXA10 Motif	pvalue
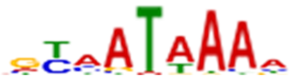	1.78e-3

Supplementary Fig.6: Validation of customized HOXA10 antibody.
Rabbits were immunized with KLH conjugated 19mer peptide of HOXA10. Four boosters were given and the sera were collected and the IgG fraction was affinity purified . These experiments were done commercially (GenScript, USA). The generated antibody was validated as below.
(A) Immunohistochemistry in paraffin sections of diestrus stage mouse endometrium. Scale bar = 100µm. Section incubated with pre-immune sera/affinity purified IgG (HOXA10 IgG)/affinity purified IgG with excess of the peptide (peptide block) used for immunization. The results revealed intense staining in the sections incubated with the HOXA10 IgG but not in the pre-immune sera. This staining was not observed in section incubated with the peptide indicating its specificity. **(B)** Immunostaining for HOXA10 in scrambled shRNA (control) and *HOXA10* shRNA (*HOXA10*KD) endometrial epithelial cells (RL95-2). We also validated the antibody in human endometrial epithelial cells, where HOXA10 was knocked down using a validated shRNA (Mishra et al, 2022) and see fig S3 and S5). Intense signals for HOXA10 were detected in the nuclei of the control cells; these signals were reduced significantly in the *HOXA10*KD cells further demonstrating its specificity. Scale bar = 100µm. **(C)** To determine if the antibody identified the HOXA10 bound to the chromatin, CUT&RUN was performed using the affinity purified HOXA10 IgG. Nuclei of RL95-2 cells treated with Estrogen and Progesterone were incubated with the IgG and CUT&RUN was performed using a commercial kit as outlined in the figure and detailed in the methodology. HOXA10 motifs was significantly enriched within the top 5k high intensity peaks.

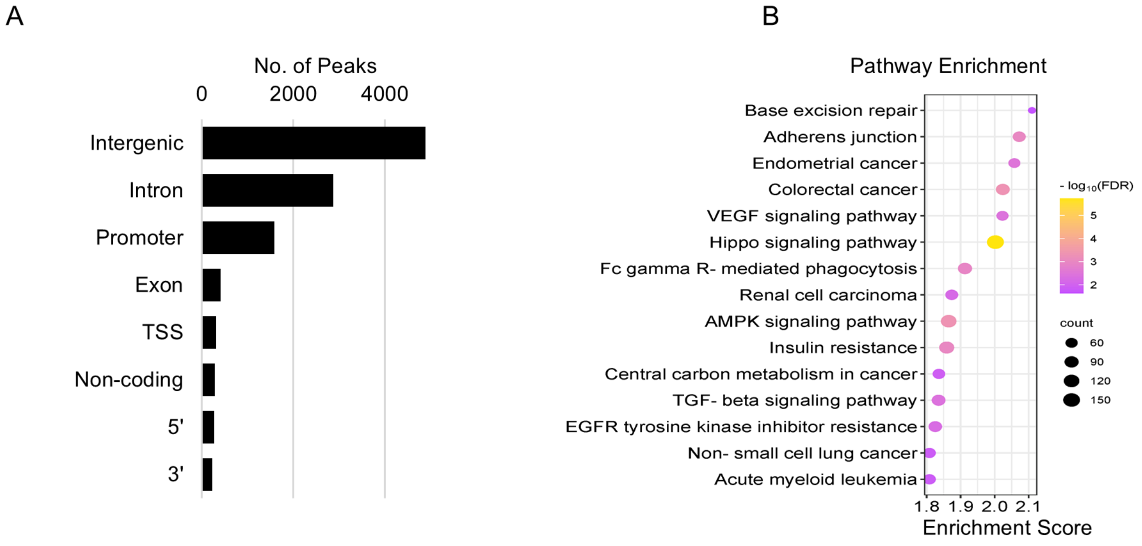

Supplementary Fig.7: Genomic localization of HOXA10 CUT&RUN peaks
(A) Location statistics of top 10,000 high confidence peaks identified from CUR&RUN for HOXA10. **(B)** Pathways associated with the genes that have HOXA10 occupancy on their promoters and transcription start site (TSS).

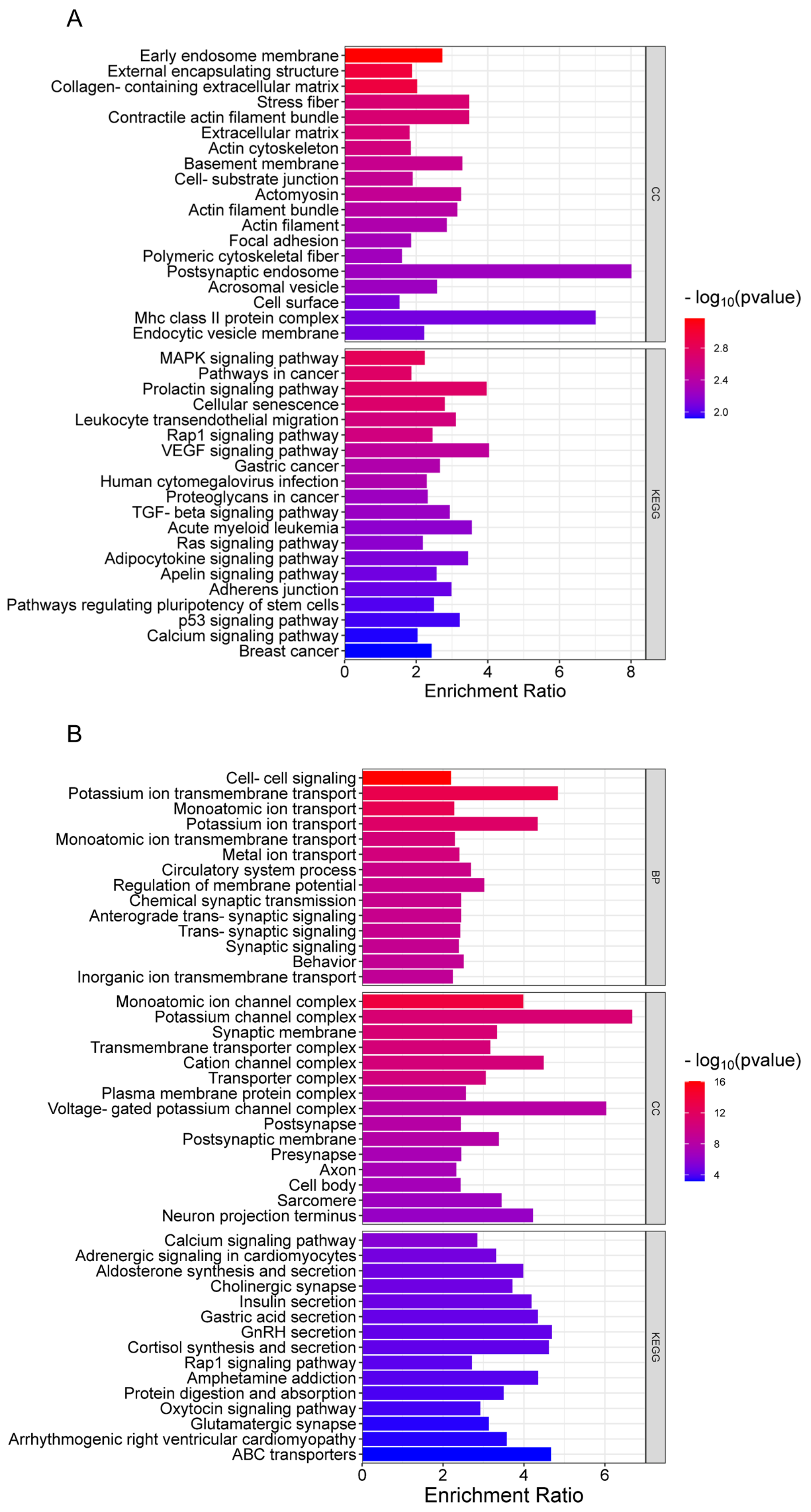

Supplementary Fig.8: Gene Ontology and Pathway Enrichment Analysis for Upregulated and Downregulated directs targets of HOXA10.

Genes that were differentially expressed in *HOXA10*KD cells and whose promoters/TSS had HOXA10 occupancy (direct targets) were subjected to GSEA. Panel representing the GO terms for **(A)** upregulated direct targets and **(B)** down regulated direct targets

A B C

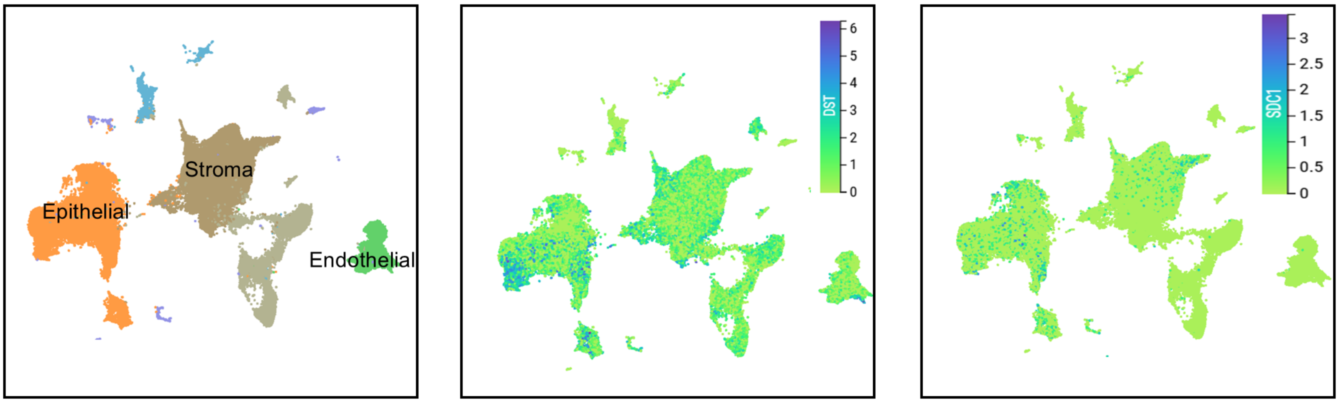

Supplementary Fig.9: Localisation of epithelial genes *DST* and *SDC1* in Human Endometrium
Data for *DST* and *SDC1* was obtained from (https://www.reproductivecellatlas.org/endometrium-all). **(A)** UMAP representations colored by cell type of human endometrium. **(B)** UMAP representations of *DST* expression in different cell type of human endometrium. **(C)** UMAP representations of *SDC1* expression in different cell type of human endometrium
